# Effect of Inhaler Design Variables on Paediatric Use of Dry Powder Inhalers

**DOI:** 10.1371/journal.pone.0099304

**Published:** 2014-06-05

**Authors:** Anne J. Lexmond, Tonnis J. Kruizinga, Paul Hagedoorn, Bart L. Rottier, Henderik W. Frijlink, Anne H. de Boer

**Affiliations:** 1 Department of Pharmaceutical Technology and Biopharmacy, University of Groningen, Groningen, The Netherlands; 2 Division of Paediatric Pulmonology and Paediatric Allergology, Beatrix Children’s Hospital, University Medical Centre Groningen, Groningen, The Netherlands; Nottingham University, United Kingdom

## Abstract

Age appropriateness is a major concern of pulmonary delivery devices, in particular of dry powder inhalers (DPIs), since their performance strongly depends on the inspiratory flow manoeuvre of the patient. Previous research on the use of DPIs by children focused mostly on specific DPIs or single inspiratory parameters. In this study, we investigated the requirements for a paediatric DPI more broadly using an instrumented test inhaler. Our primary aim was to assess the impact of airflow resistance on children’s inspiratory flow profiles. Additionally, we investigated children’s preferences for airflow resistance and mouthpiece design and how these relate to what may be most suitable for them. We tested 98 children (aged 4.7–12.6 years), of whom 91 were able to perform one or more correct inhalations through the test inhaler. We recorded flow profiles at five airflow resistances ranging from 0.025 to 0.055 kPa^0.5^.min.L^−1^ and computed various inspiratory flow parameters from these recordings. A sinuscope was used to observe any obstructions in the oral cavity during inhalation. 256 flow profiles were included for analysis. We found that both airflow resistance and the children’s characteristics affect the inspiratory parameters. Our data suggest that a medium-high resistance is both suitable for and well appreciated by children aged 5–12 years. High incidences (up to 90%) of obstructions were found, which may restrict the use of DPIs by children. However, an oblong mouthpiece that was preferred the most appeared to positively affect the passageway through the oral cavity. To accommodate children from the age of 5 years onwards, a DPI should deliver a sufficiently high fine particle dose within an inhaled volume of 0.5 L and at a peak inspiratory flow rate of 25–40 L.min^−1^. We recommend taking these requirements into account for future paediatric inhaler development.

## Introduction

Drug delivery to the lungs is complex and involves several process steps depending on the inhalation device used. To achieve sufficient pulmonary deposition, the inhalation device has to be prepared and operated correctly. Particularly for dry powder inhalers (DPIs), the inhalation manoeuvre is of utmost importance, as it determines both the fine particle dose delivered and the site of deposition of the particles in the lungs [1]. For most marketed DPIs, the energy for releasing and dispersing the powder into an aerosol with a proper aerodynamic particle size distribution is derived from the inhaled air stream through the inhaler. To have sufficient energy available, the airflow rate has to exceed a certain threshold value, which is dependent on the inhaler design, and the inhaled volume has to be sufficiently large for transport of the aerosol into the target area [2]. Additionally, a breath hold period after inhalation is desired to give particles sufficient time for sedimentation in the central and peripheral airways [1].

The ability to perform an inhalation manoeuvre that complies with the requirements for good performance of a particular type of DPI depends on the clinical picture (*i.e.* disease severity) and age of the patient, due to physical limitations and improper understanding of how to handle the device [3]–[6]. Children comprise an important target population for inhalation therapy, but limited fundamental research has been done on their cognitive and inspiratory capacities to operate dry powder inhalers (DPIs). Most studies on dry powder inhalation in children focused either on their ability to operate a specific DPI [7]–[13], or on single inspiratory parameters, especially the peak flow rate [14]–[17], and how these are affected by the airflow resistance of the inhaler [18]–[21]. Moreover, many peak flow studies were performed with the In-Check Dial [16], [18]–[20], a device that mimics the airflow resistance of some marketed inhalers, but does not take into account other possible constraints like inhaled volume.

The primary aim of our study was to assess the impact of airflow resistance on the inspiratory flow profiles that school children can generate by use of a test inhaler with exchangeable airflow resistance. Our secondary aims were to investigate the children’s preferences for airflow resistance and mouthpiece design and how these preferences relate to what may be most suitable for them. A sinuscope was inserted into the test inhaler in order to acquire videos of the oral cavity during inhalation for assessment of the impact of resistance and mouthpiece design on the geometry of the oral cavity during inhalation.

## Subjects and Methods

### Subjects

All children (4–13 years of age) from a primary school in the Groningen city area (The Netherlands) with written informed consent from their parents or guardians were eligible to participate voluntarily in the study. No exclusion criteria were applied, although annotations were made for children with rhinitis or diagnosed airways disease. The study was approved by the Medical Ethics Committee of the University Medical Centre Groningen.

### Test Inhaler

**Figure 1:**
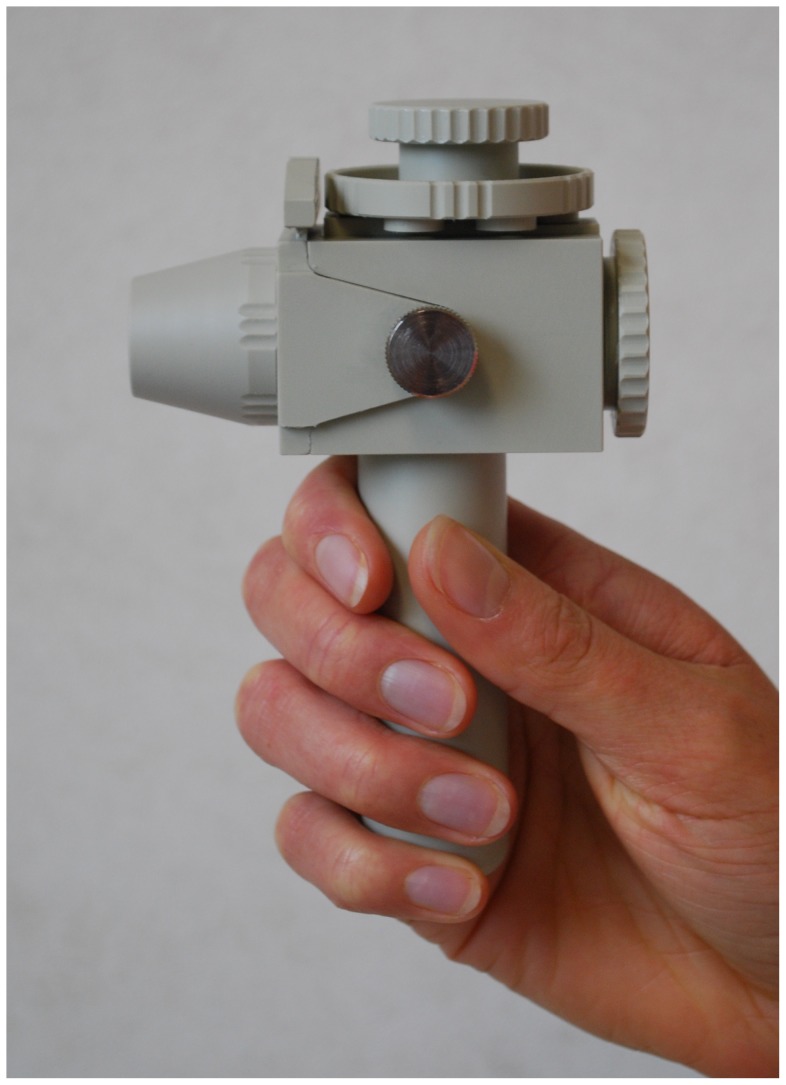
The test inhaler with exchangeable airflow resistance (disk on top) and the conical mouthpiece.

**Figure 2:**
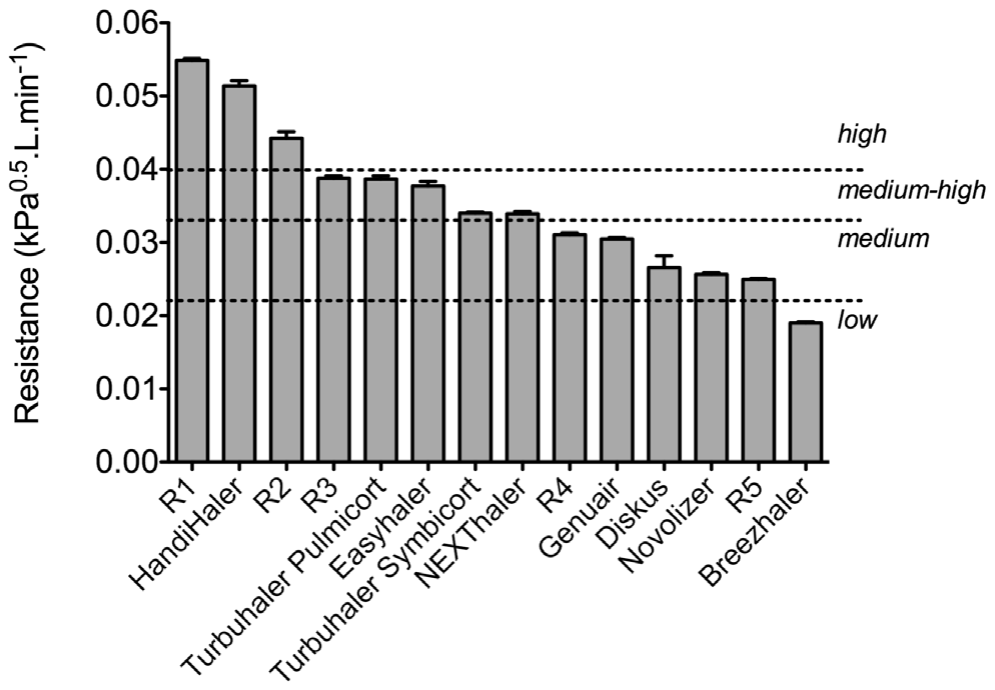
The resistance modes of the test inhaler. Resistance modes (R1–R5) are shown in comparison to the airflow resistances of commercial DPIs, as measured with the same equipment used for the study. Shown are the mean resistance and SEM determined on five test inhalers (at least five data points per resistance mode) and on at least three DPIs (nine data points per inhaler). The dashed lines represent the resistance classes according to the ERS/ISAM Task Force [22].

**Figure 3:**
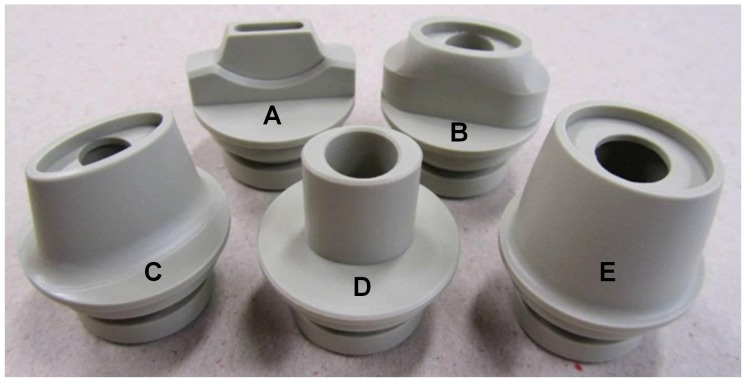
The five additional mouthpiece designs for the test inhaler. A) flat terraced; B) oblong terraced; C) oblong; D) oval small; E) oval large.

A dummy test inhaler with exchangeable mouthpieces and airflow resistances was designed for the study (Figure 1). The resistance was controlled with a rotatable ring with differently sized orifices in front of the inlet channel covering a range from 0.025 to 0.055 kPa^0.5^.min.L^−1^, or from medium to high according to the definitions of the ERS/ISAM Task Force (Figure 2) [22]. The mouthpiece designs were based on marketed and investigational DPIs, including the Twincer (A), Diskus (B) HandiHaler (C), Cyclohaler or Aerolizer (D), and Novolizer (E) (Figure 3). The test inhaler was connected to a differential pressure gauge (HBM, The Netherlands), which measured the pressure drop across the inhaler during inhalation. The differential pressure gauge was linked to a computer programmed to calculate the inspiratory flow rate as function of the inhalation time (the flow profile) from the measured pressure drop, using previously recorded flow rate–pressure drop relationships for the individual airflow resistances (Labview software, National Instruments BV, The Netherlands). The flow profile was displayed on the computer screen allowing for visual feedback during the inhalations. A sinuscope (Olympus WA96200A, Olympus Winter & Ibe GmbH, Germany) with its optics near the front opening of the mouthpiece was used to observe the oral cavity. The sinuscope was inserted from the (otherwise closed-off) rear end of the test inhaler and was air-tightly secured to prevent air leakage that would affect the airflow resistance of the inhaler. The test inhaler was mounted on a freely movable stand and counterbalanced to relieve its weight.

### Study Design

**Figure 4:**
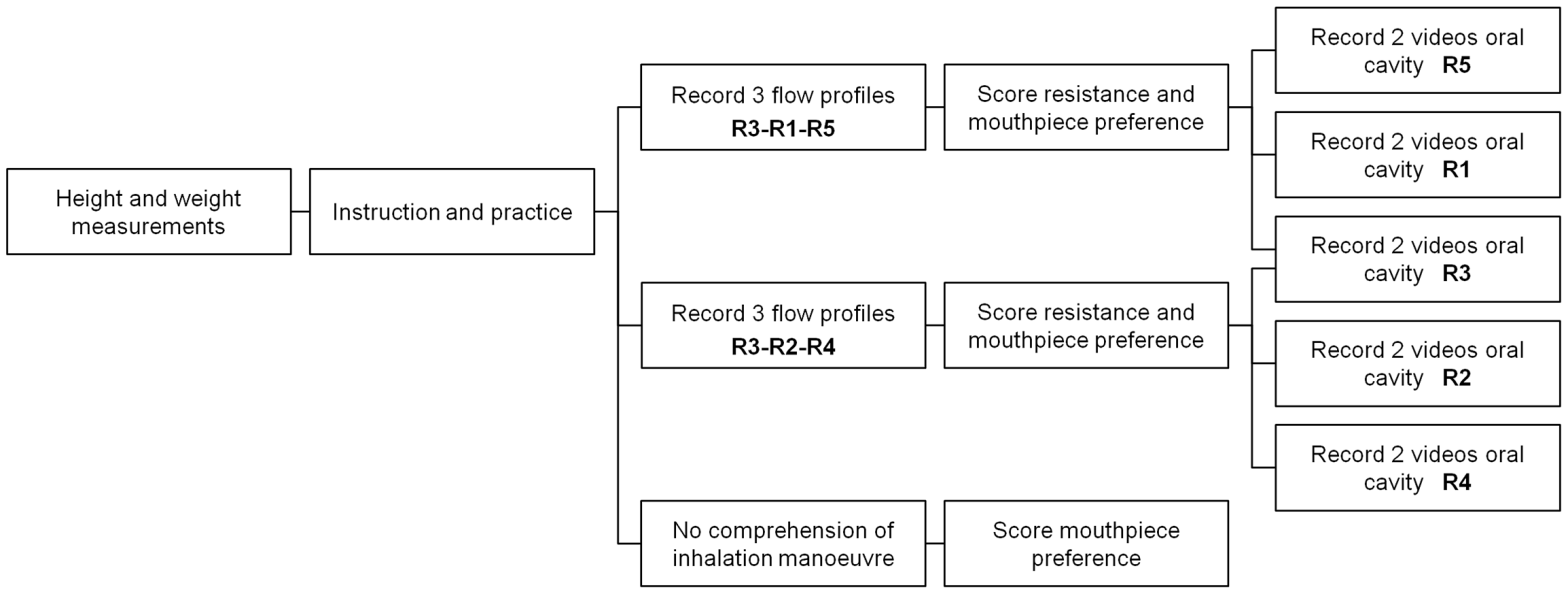
Flow diagram of the test procedures.

The study had an exploratory, non-therapeutic, observational design. The children were tested individually (4–5 years old) or in pairs (≥6 years). In Figure 4, a flow diagram of the study procedures is given, which were completed on one occasion per child. Assignment of the resistance regimen (R3-R1-R5 or R3-R2-R4) was based on the number of enrolment.

First, three flow profiles with different airflow resistances were recorded using a conical mouthpiece (shown in Figure 1). Subsequently, the children were asked to indicate which of the three resistances they preferred most and to choose their favourite mouthpiece from five additional designs (Figure 3). Children who performed exhalations rather than inhalations during these measurements were considered ineligible to participate in the final procedures of the study. The eligible children were asked to do two more inhalations using the conical mouthpiece and their mouthpiece of choice (both with preferred airflow resistance), during which the oral cavity was recorded by camera.

### Flow Profiles

The children were instructed to stand up straight, hold the inhaler in the correct position (away from the mouth) and exhale completely (not through the inhaler) before bringing the inhaler in position for the inhalation. They were also instructed how to place the mouthpiece between the lips, to inhale as strongly and as long as possible, take the inhaler from the mouth and hold their breath for as long as comfortable (up to 10 s), and finally to exhale. Following the instructions, the children were given the opportunity to practice the procedure whilst seeing their flow profile on a monitor and receiving feedback from the instructor, after which they were asked to repeat the instructions either verbally or by demonstration. Next, the three flow profiles were recorded using a medium-high (R3), a higher (R1 or R2), and a lower (R4 or R5) resistance (Figure 2).

During inhalation, compliance with all different aspects of the instruction was scored to assess the usefulness of the flow profiles. Immediate exclusion from further processing followed when exhalation through the test inhaler was demonstrated. Flow profiles were quarantined first in case of sub-maximal scores regarding compliance or discontinued inhalation and were evaluated separately afterwards. Secondary exclusion followed from scoring three points or more from the events shown in Table 1. The events: insufficient pressure drop, inhaled volume, and inhalation time contributed to the total score, but flow profiles were not excluded exclusively on these events together, as this could also imply that a child was not able to perform better.

**Table 1:** Events evaluated for secondary exclusion of flow profiles.

Event	Points
The child reported the effort being too high	3
No exhalation prior to inhalation	2
Exhalation through inhaler prior to inhalation	2
The child reported having a cold	1
Maximum pressure drop<1 kPa*	1
Total inhaled volume <0.5 L*	1
Total inhalation time <0.5 s*	1

Secondary exclusion when total score ≥3 points.

*No exclusion based exclusively on these events together.

Characteristic inspiratory parameters computed from accepted flow profiles were the peak flow rate (PIF), flow increase rate (mean acceleration in flow rate between 20% and 80% of PIF; FIR_20–80%_) [23], inhaled volume (V_i_), and total inhalation time (t_i_).

### Oral Cavity Videos

During the two final inhalations with the conical and the preferred mouthpiece, the oral cavity was recorded by video camera to observe whether obstructions were present in the passageway for an aerosol during inhalation and whether the occurrence of obstructions depended on the airflow resistance and mouthpiece design. The videos were evaluated qualitatively by two investigators independently. Obstructions were considered to be present when it was estimated that less than a third of the throat opening was visible, when the tongue was raised, or the cheeks were curved inwards during inhalation. The incidence of these events was evaluated during four 0.5 s time intervals and expressed as a percentage of the total number of recorded videos per resistance mode and mouthpiece design. Other unwanted events, *e.g.* seeing teeth in front of the mouthpiece opening, were noted separately.

### Data Analysis

Exploratory data analysis was performed as a first approach to identify data distribution trends. Normality was tested using Shapiro-Wilk normality test. Pearson’s (for normally distributed variables) or Spearman’s (for non-normally distributed variables) correlation coefficients were calculated to study the correlation between the inspiratory parameters and age. Subsequently, linear mixed models were used to estimate the effects of airflow resistance and the children’s characteristics on the inspiratory parameters. The presence of obstructions in the oral cavity and the effects of mouthpiece design and airflow resistance thereon were evaluated qualitatively. Analyses were performed with SPSS 20 (IBM) and Prism 5.0 (GraphPad Software). All statistical testing was two-sided, with an α of 0.05.

## Results

### Subjects

104 children were recruited for the study. One child was ill on the test day and five dropped out during the test procedure, leaving 98 children (age range: 4.7–12.6 years) who completed the entire test procedure, of whom 91 performed at least one correct inhalation manoeuvre. The profiles of four children, who were all younger than 6 years of age, were immediately excluded because they exhaled through the test inhaler. Age, height, weight, and gender distributions of the 91 children are shown in Table 2. Five of the children had a diagnosed airways disease (three asthma, two CF; 7.3–11.8 years) and thirteen children reported having a cold or runny nose. The children with a diagnosed airways disease had no apparent acute symptoms.

**Table 2:** Descriptive statistics of the children who performed one or more correct inhalations (*N*  = 91).

	Mean	Median	SD	Range
Age (years)	9.2	9.5	1.9	5.3–12.6
Height (cm)	140.0	140.0	13.1	108.5–173.0
Weight (kg)	33.3	32.6	8.4	16.0–55.6
Gender	Male: 40 (44%)		Female: 51 (56%)	

### Flow Profiles

In total, 256 correct flow profiles from 91 children were accepted for analysis. Of 16 children, one or more flow profiles were rejected based on the various exclusion criteria defined in the *Subjects and methods* section. Table 3 presents a summary of the computed data for the four parameters per resistance. PIF and V_i_ were found to increase significantly with age. For all resistances, strong positive correlations exist between V_i_ (*p*<0.0001) respectively PIF (*p*<0.001) and age. For t_i_, the increases with age are less pronounced and not statistically significant for two out of five resistances; for FIR_20–80%_, an overall significant increase was found, but not for the separate resistance modes.

**Table 3:** Correlation analysis between the inspiratory parameters and the children’s age.

PIF (L.min^−1^)	*N*	Mean	SD	*r*	95% CI
*Total*	*256*	*49*	*17.5*	.*47*	*[.37,.57]*
R1	44	37	9.0	.54	[.28,.72]
R2	42	43	12.9	.63	[.40,.78]
R3	85	46	13.7	.64	[.49,.75]
R4	41	57	17.7	.53	[.27,.72]
R5	44	66	18.0	.44	[.16,.66]
**FIR (L.s^2^)**	***N***	**Mean**	**SD**	***r***	**95% CI**
***Total***	***256***	***2.17***	***1.911***	**.** ***20***	***[.073,.32]***
R1	44	1.60	1.189	.21	[−.10,.48]
R2	42	1.74	.959	.28	[−.033,.55]
R3	85	2.04	2.112	.12	[−.10,.33]
R4	41	2.32	1.807	.34	[.027,.59]
R5	44	3.27	2.409	.07	[−.24,.37]
**V_i_ (L)**	***N***	**Mean**	**SD**	***r***	**95% CI**
***Total***	***256***	***1.30***	**.** ***514***	**.** ***68***	***[.61,.74]***
R1	44	1.20	.465	.65	[.43,.80]
R2	42	1.31	.502	.72	[.54,.84]
R3	85	1.27	.555	.71	[.58,.80]
R4	41	1.41	.470	.73	[.55,.85]
R5	44	1.35	.523	.69	[.49,.82]
**t_i_ (s)**	***N***	**Mean**	**SD**	***r***	**95% CI**
***Total***	***256***	***2.40***	**.** ***771***	**.** ***30***	***[.18,.41]***
R1	44	2.80	.860	.30	[−.0087,.55]
R2	42	2.68	.685	.36	[.066,.60]
R3	85	2.42	.690	.41	[.21,.57]
R4	41	2.27	.715	.24	[−.086,.51]
R5	44	1.82	.568	.46	[.18,.67]

**Figure 5:**
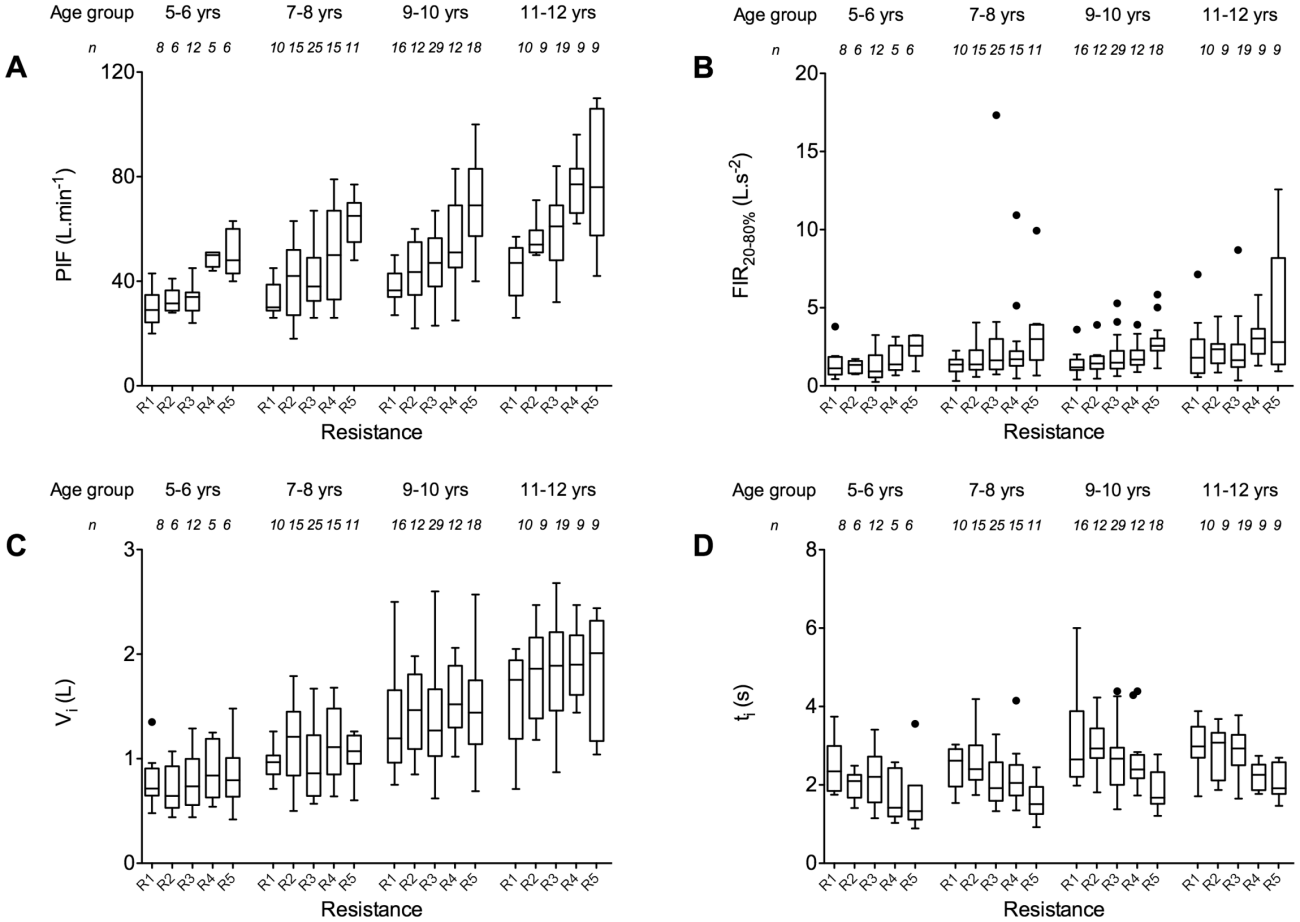
Box-whisker plots of the inspiratory data grouped by age and airflow resistance. A) peak inspiratory flow rate (PIF); B) mean acceleration in airflow from 20% to 80% of the peak inspiratory flow rate (flow increase rate: FIR_20–80%_); C) inhaled volume (V_i_); D) inhalation time (t_i_). The boxes range from the lower to the upper quartiles, within which the median is indicated. The whiskers present the lowest datum still within 1.5 times the interquartile range (IQR) of the lower quartile, and the highest datum within 1.5 IQR of the upper quartile. Outliers (•) are defined as any value that lays more than 1.5 IQR from either end of the box. The dataset can be found in the Supporting Information (Data S1).

Figure 5 shows the computed values for the inspiratory parameters per resistance per age group. Figure 5A illustrates how mean PIF (as well as its spread) increases with decreasing resistance for all groups. Individual minimum PIF values vary from 20 L.min^−1^ for the highest (R1 or R2) to 40 L.min^−1^ for the lowest (R4 and R5) resistances. Also FIR_20–80%_ increases with decreasing resistance, but several extreme values were recorded for this parameter and the trends are considerably less clear than for PIF (Figure 5B). When using the lowest resistance, the oldest age group performed quite variably regarding FIR_20–80%_. No consistent effect of airflow resistance on inhaled volume was apparent (Figure 5C, all age groups). For the inhalation time, a decreasing trend exists with decreasing resistance, similar for all age groups (Figure 5D). When using the lowest resistance, minimum values of around 1 s inhalation time were recorded, whereas at the highest two resistances, all inhalations lasted at least 1.5 s.

Although Figure 5 provides an illustrative summary of all the computed data for the inspiratory parameters, it does not truly depict the effect of resistance on these parameters. Other characteristics of the children that affect their performance have to be taken into account as well. It is well known that besides age, also gender and height determine the inspiratory capacities of children [24]. To that end, we modelled the inspiratory parameters for children aged 5–12 years with the resistance modes, age, height, gender, and a factor for airways disease as covariates (Table 4). Since height and age are strongly correlated (simple linear regression analysis yielded an *R* ^2^ of 0.804), we included a variable for the residual variation in height rather than height itself. The resistance modes were now found to affect all inspiratory parameters, including V_i_. Interestingly, we distinguished a positive effect of the presence of an airways disease on PIF, FIR_20–80%_, and V_i_. The fact that this was the only variable besides resistance that affected FIR_20–80%_ may indicate that their better performance is likely due to the children’s familiarity with performing inhalation manoeuvres.

**Table 4:** Linear mixed model parameter estimates of the effects of airflow resistance and the children’s characteristics on the inspiratory parameters.

	PIF (L.min^−1^)		FIR_20–80%_ (L.s^-2^)		V_i_ (L)		t_i_ (s)	
	Parameter estimate ± SE	*p*-value	Parameter estimate ± SE	*p*-value	Parameter estimate ± SE	*p*-value	Parameter estimate ± SE	*p*-value
Intercept	31±5.6	<.0001	2.7±.80	.001	−.19±.16	.223	.81±.30	.009
R1^a^	−30±1.4	<.0001	−1.7±.25	<.0001	−.18±.034	<.0001	.91±.079	<.0001
R2^a^	−24±1.7	<.0001	−1.6±.31	<.0001	−.11±.044	.014	.80±.10	<.0001
R3^a^	−21±1.3	<.0001	−1.2±.24	<.0001	−.13±.033	.0001	.55±.075	<.0001
R4^a^	−10±1.8	<.0001	−1.0±.32	.001	−.030±.045	.506	.37±.10	.0003
Gender^b^	−5.6±2.2	.014	−.38±.31	.218	−.25±.062	.0001	−.093±.12	.443
Airways disease^c^	9.7±4.8	.047	2.4±.66	.001	.45±.13	.001	.46±.26	.077
Age (years)	4.1±.58	<.0001	.077±.081	.343	.18±.016	<.0001	.12±.031	.0004
Height_resid_ d (cm)	.072±.189	.706	.018±.026	.497	.015±.0053	.006	.027±.010	.010
Random intercept	93±16		1.5±.30		.075±.012		.26±.046	
Residual	38±4.2		1.3±.15		.024±.0026		.13±.014	
*N*	256		256		256		256	

PIF: peak inspiratory flow rate; FIR_20–80%_: flow increase rate; V_i_: inhaled volume; t_i_: inhalation time.

a Reference category is R5.

b Reference category is male.

c Reference category is no airways disease.

d Height_resid_ = Height – Height_exp_. Height_exp_ obtained by linear regression: Height_exp_  = 6.152 *Age +83.362 (adjusted *R* ^2^ = .804).

The dataset can be found in the Supporting Information (Data S1).

### Breath Hold Time

Of the 91 children who performed at least one correct inhalation, the majority (74%) held their breath for at least three seconds after the inhalation, and 47% could extend the breath hold time to more than six seconds. Three children did not hold their breath at all, even not upon repeated practicing.

### Children’s Preferences for Airflow Resistance and Mouthpiece Design

**Figure 6:**
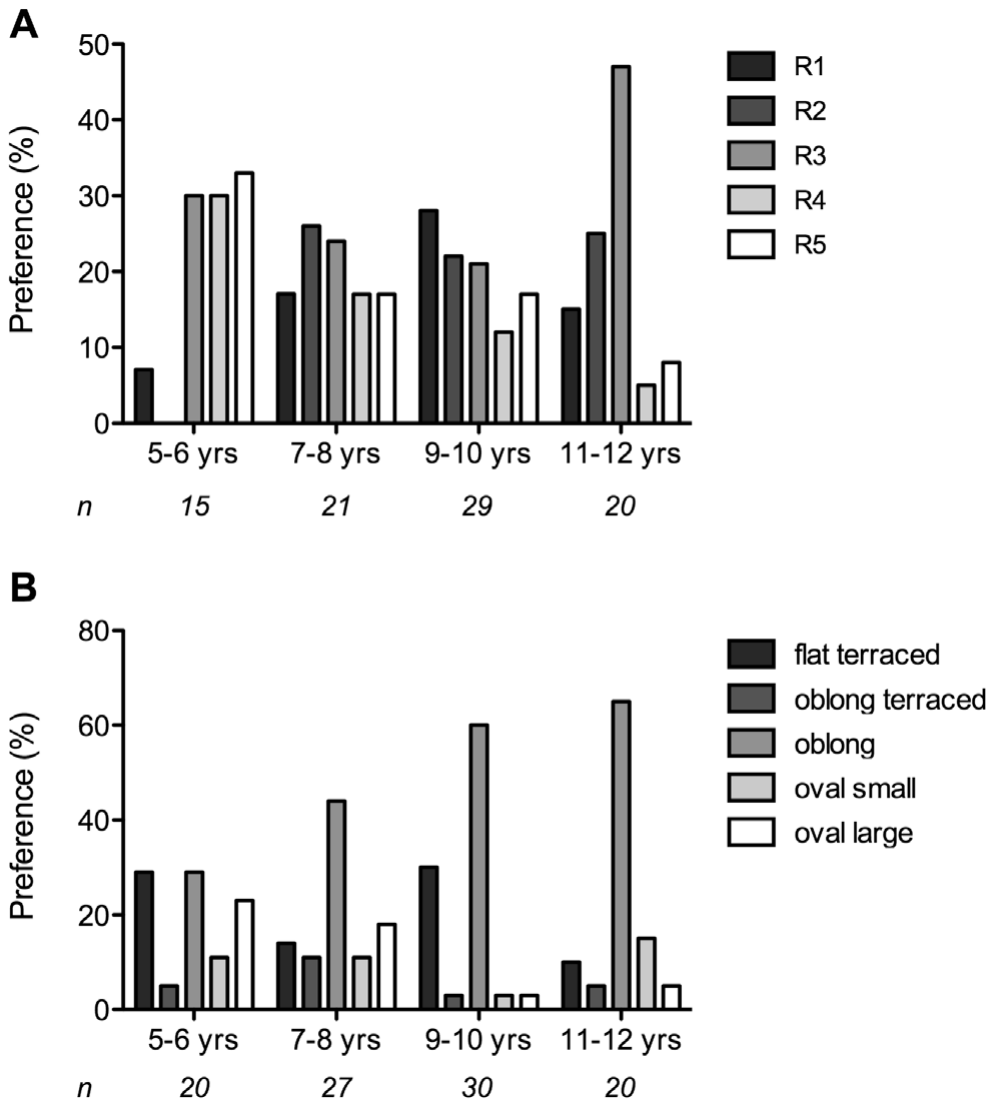
Children’s preferences in percentages per age group. A) airflow resistance (*N*  = 85); B) mouthpiece design (*N*  = 97).

Most children in the age group 5–6 years preferred the lower resistance modes (R3–R5) (Figure 6A). The children in the other age groups had a stronger preference for a medium to high resistance (R1–R3) and the preference became more pronounced with increasing age. Eight children did not express preference for any of the resistances.

Figure 6B shows that most children preferred an oblong mouthpiece design (on average 51% for all age groups). This preference became more pronounced with increasing age. In the youngest age group, preferences were much more diverse than in the older groups.

### Oral Cavity Recordings

A total of 184 videos from 94 children were analysed on obstructions in the oral cavity during inhalation. Table 5 summarises how often the different events – as defined in the *Subjects and methods* section – were observed in subsequent time intervals per resistance mode, when the conical mouthpiece was used. Poor visibility of the throat and a raised position of the tongue were observed frequently and in approximately the same percentage of cases for all five resistance modes. Only the incidence of inwards curvature of the cheeks appeared to increase with increasing resistance. For all three events, the occurrence decreased in time, most likely as a result of relaxation of the mouth and throat region.

**Table 5:** Incidences of the events observed during the inhalation manoeuvres with the conical mouthpiece per resistance mode.

			Incidence (% of total number of videos per resistance mode)													
		Meanage	Throat visibility <33%				Tongue raised				Cheeks inwards				Condensation	
	*N*	(years)	0–0.5 s	0.5–1 s	1–1.5 s	>1.5 s	0–0.5 s	0.5–1 s	1–1.5 s	>1.5 s	0–0.5 s	0.5–1 s	1–1.5 s	>1.5 s	begin	end
R1	19	9.6	79	63	50	29	63	55	34	23	53	53	39	3	5	26
R2	25	9.4	90	80	74	39	76	66	66	30	38	44	36	0	4	44
R3	23	9.7	78	69	50	26	74	51	41	26	41	29	20	3	4	30
R4	11	8.2	82	64	45	40	59	50	45	35	32	32	14	0	0	73
R5	14	8.6	75	64	39	39	64	39	25	29	29	32	18	0	14	43

The dataset can be found in the Supporting Information (Data S2).

The influence of the different mouthpiece designs on the occurrence of the three types of obstructions is shown in Table 6. In this table, only the events observed during the time interval 0.5–1 s are shown for clarity. We chose this interval because in the first interval placement of the inhaler may affect the data, whilst from 1 s onwards relaxation of the mouth may already occur. The groups are not well comparable due to differences in number of observations, the airflow resistance, and the age of the children who used the mouthpiece and therefore, these data are only indicative. The smaller designs (flat terraced, oblong, oval small) appeared to have a positive effect on the position of the cheeks. The oval large mouthpiece, which was the largest design we tested, seemed to positively affect the positioning of the inhaler in the mouth (throat visibility) and it also helped in keeping the tongue down. However, the children who chose this design were younger than those in the other groups. Therefore, the effects may partly be due to age (*i.e.* less forceful inhalations).

**Table 6:** Incidences of the events observed during the inhalation manoeuvres per mouthpiece design.

				Incidence (% of total number of videos per mouthpiece design)				
		Mean age	Mean R	Throat vis <33%	Tongue raised	Cheeks inwards	Condensation	
	*N*	(years)		0.5–1 s	0.5–1 s	0.5–1 s	begin	end
Conical	92	9.2	2.7	69	54	39	4	40
Flat terraced (A)	19	8.8	2.5	68	32	16	5	53
Oblong terraced (B)	6	8.8	2.7	92	58	50	0	83
Oblong (C)	46	9.6	2.7	59	42	20	15	48
Oval small (D)	9	9.1	3.1	72	61	11	6	61
Oval large (E)	12	7.9	3.0	33	33	42	17	17

The dataset can be found in the Supporting Information (Data S2).

**Figure 7:**
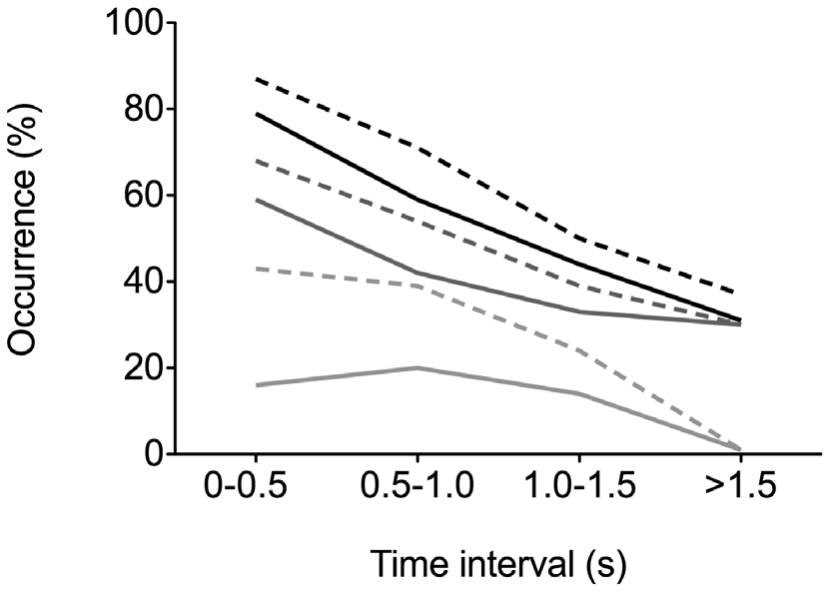
Comparison of the conical and oblong mouthpieces in terms of the occurrence of obstructions. The occurrence of obstructions is shown for subsequent 0.5(dashed lines) and oblong (solid lines) mouthpiece (*N*  = 46). The number of data decreases with increasing time interval because not all children extended their inhalation towards >1.5 s. Throat vis <33%: less than a third of the throat opening is visible; Tongue raised: the tongue is raised from the base of the mouth; Cheeks inwards: the cheeks are curved inwards and clearly visible on the video.

Since half of the children chose the oblong mouthpiece, a considerable amount of data is available to evaluate the effects of this mouthpiece design on the incidence of the various events. In Figure 7, the incidence of the three events is plotted as trend over inhalation time for the subgroup of 46 children who used the oblong mouthpiece, in comparison to the conical mouthpiece. This figure suggests that this most preferred mouthpiece might reduce all types of obstructions.

Another observation with the sinuscope was that 44% of all inhalations ended with condensation of moisture on the lens, which can most likely be attributed to exhaling. Also, in 8% of all inhalations, we observed moisture condensation preceding the inhalation. In Tables 5 and 6, the occurrence of such condensation is shown per resistance mode and mouthpiece design respectively. Particular events observed with the sinuscope were: teeth in front of the orifice (seven times), lips in front of the orifice (ten times), both teeth and lips in front of the orifice (ten times), pointing the inhaler downwards (seven times), and pointing the inhaler towards the side (one time).

## Discussion

In this study, we investigated the applicability of dry powder inhalation in school children by a general (non-inhaler specific) approach using an instrumented test inhaler. The most encouraging finding of this study is that nearly all (91) children (equals 93%) were able to perform at least one inhalation correctly, the youngest being 5.3 years of age. The inspiratory parameters PIF, V_i_, and t_i_ are positively correlated to the children’s age because of their dependence on total lung capacity. The parameters are interrelated, which explains the differences in trends (Figure 5). When the resistance is increased, the same inspiratory effort results in a higher pressure drop, but the increase is not to such an extent that the flow rate remains the same [23]. Therefore, the maximally attainable flow rate decreases with increasing resistance. As the lungs fill up more slowly at a lower flow rate, inhalation takes longer through a higher resistance. However, there are limitations to the duration of inhalation manoeuvres, which for a DPI may be problematic when the inhaled volume becomes insufficient for aerosol transport into the distal airways. The effect of airflow resistance on inhaled volume that we found in this study implies that there are limitations to the resistance of a paediatric DPI. A difference of almost 0.2 L was estimated between the inhaled volumes at the lowest and the highest resistance. Especially for children aged 5 to 7 years, for whom all inhaled volume estimates are less than 1 L, a high resistance (R1 and R2 in this study) may not be appropriate. The positive effect of having an airways disease on the inspiratory parameters (except t_i_) suggests that by training, a child’s inspiratory capacity can be improved. Since our study included only five children with airways disease, the effects of training will be further investigated in a follow-up study in children who are experienced with inhalation therapy.

A limitation of this study is the low number of children younger than 7 years, which is the most critical age group, especially regarding the understanding of the procedure. Only two 4-year olds finished the study, and both of them did not comprehend the inhalation procedure. Two out of four 5-year olds (50%) were capable of performing a correct inhalation manoeuvre versus 14 out of 15 6-year olds (93%), which is in line with percentages reported before [6]. Furthermore, it should be emphasised that we have not investigated the repeatability and sustainability of the children’s inhalation technique. Our aim was to characterise what children can maximally achieve, which can serve as a starting point for device design. Hence, our models provide estimates for the inspiratory parameters that can be used when developing a DPI for children from the age of 5 years onwards.

Applying a breath hold period, which is important for sedimentation deposition of aerosol particles in the airways, does not appear to be an important constraint for DPI use by children. On the contrary, the presence of obstructions in the oral cavity, which we observed especially in the first second of the inhalation (Table 5, Figure 7), can present a serious restriction. The true implications of these observations are not clear yet, since we have no reference as to whether the obstructions that we defined would actually impede an aerosol. To this end, deposition studies may be required, which are not easily conducted in paediatric subjects. Our findings do suggest that mouthpiece design rather than airflow resistance affects the geometry of the oral cavity upon inhalation. The overall preference for an oblong mouthpiece is therefore fortunate, as this design appears to have a positive effect on the passageway for the aerosol through the mouth. This effect may possibly be enlarged by further optimisation of the mouthpiece design.

Another concern is the moisture condensation on the sinuscope lens observed at the end of the inhalation with a much higher incidence than the incidence of exhalations reported by the observing investigator (44% and 10% respectively). The condensation may, at least in a part of the cases, be the result of the environmental change in the oral cavity that occurs after the inhalation has stopped rather than the result of a true exhalation. Still, this finding implies that even when a child finishes the procedure properly to the eye, (some) wetting of the inside of the inhaler may occur.

Preference for resistance depends on age, but as a fair compromise, a medium-high [22] resistance seems well acceptable for all age groups. The resistance of an inhaler could be an aspect that a patient learns to appreciate over time. Most children in our study were naive to inhalation therapy and it would be interesting to study how experience influences a child’s perception of what is comfortable.

In conclusion, the large dataset and models of inspiratory capacities of school children obtained with this study are helpful for designing a DPI that is suitable for children (older than 5 years) without inspiratory restrictions. Specifications of such an inhaler are that the entire dose is released within 0.5 L of inhaled air and that dispersion of the drug formulation is satisfactory at a peak inspiratory flow rate of 25 to 40 L/min through a medium-high to medium resistance inhaler. Most currently marketed inhalers do not meet these requirements and children using them risk under-dosing. For companies seeking to develop DPI products for the paediatric population, assessing the dose emission profile of the DPI using the flow profiles we obtained in this study may be a useful development tool. An inhaler for children can have a medium-high resistance, as it is both acceptable for children and beneficial for increasing the duration of the inhalation without compromising inhaled volume too much. Additionally, a paediatric DPI should be easy to handle and preferably have an oblong mouthpiece. Lastly, suitable means to protect the inhaler’s interior against exhalation through the device are strongly desired.

## Supporting Information

Data S1 **Dataset of the children’s characteristics and inspiratory parameters.**
(XLSX)

Data S2 **Dataset of the video analysis.**
(XLSX)
